# Targeting Microglia in Alzheimer’s Disease: Pathogenesis and Potential Therapeutic Strategies

**DOI:** 10.3390/biom14070833

**Published:** 2024-07-11

**Authors:** Zhongqing Sun, Xin Zhang, Kwok-Fai So, Wen Jiang, Kin Chiu

**Affiliations:** 1 Department of Neurology, Xijing Hospital, Fourth Military Medical University, Xi’an 710032, China; 2 Department of Ophthalmology, School of Clinical Medicine, Li Kai Shing Faculty of Medicine, The University of Hong Kong, Hong Kong SAR, China; 3 State Key Lab of Brain and Cognitive Sciences, Li Kai Shing Faculty of Medicine, The University of Hong Kong, Hong Kong SAR, China; 4 Guangdong-Hongkong-Macau Institute of CNS Regeneration, Key Laboratory of CNS Regeneration (Ministry of Education), Jinan University, Guangzhou 510632, China; 5 Department of Psychology, The University of Hong Kong, Hong Kong SAR, China

**Keywords:** Alzheimer’s disease, microglia, Aβ deposition, neuroinflammation, microglia-related targets, nature products, phagocytosis

## Abstract

Microglia, as resident macrophages in the central nervous system, play a multifunctional role in the pathogenesis of Alzheimer’s disease (AD). Their clustering around amyloid-β (Aβ) deposits is a core pathological feature of AD. Recent advances in single-cell RNA sequencing (scRNA-seq) and single-nucleus RNA sequencing (snRNA-seq) have revealed dynamic changes in microglial phenotypes over time and across different brain regions during aging and AD progression. As AD advances, microglia primarily exhibit impaired phagocytosis of Aβ and tau, along with the release of pro-inflammatory cytokines that damage synapses and neurons. Targeting microglia has emerged as a potential therapeutic approach for AD. Treatment strategies involving microglia can be broadly categorized into two aspects: (1) enhancing microglial function: This involves augmenting their phagocytic ability against Aβ and cellular debris and (2) mitigating neuroinflammation: Strategies include inhibiting TNF-α signaling to reduce the neuroinflammatory response triggered by microglia. Clinical trials exploring microglia-related approaches for AD treatment have garnered attention. Additionally, natural products show promise in enhancing beneficial effects and suppressing inflammatory responses. Clarifying microglial dynamics, understanding their roles, and exploring novel therapeutic approaches will advance our fight against AD.

## 1. Introduction

Alzheimer’s disease (AD) is the predominant cause of dementia worldwide, and the number of patients with dementia is estimated to reach 75 million by 2030 [1]. AD is a multifactorial, heterogeneous, and progressive neurodegenerative disease characterized by a lengthy pre-clinical phase. Amyloid-β (Aβ)-proteopathy is the crucial early impetus for the disease [2,3,4]. The FDA’s approval of several anti-Aβ drugs (Aducanumab and Lecanemab) for clinical use provides significant achievements for both basic research and clinical investigations on AD [5].The pathological hallmarks of AD in the brain are extracellular Aβ plaques, hyperphosphorylated tau-formed neurofibrillary tangles, and significant neuron loss [6,7]. Large-scale genome-wide association studies (GWASs) have identified the AD risk genes associated with innate immune functions as well as elevated levels of inflammatory cytokines in AD patients, which suggests that neuroinflammation plays a prominent role in the pathology of AD [8,9,10]. The latest Bayesian genome-wide (BGW) transcriptome-wide association study (TWAS) highlighted the critical involvement of apolipoprotein C 2 (*APOC2*), bridging integrator 1 (*BIN1*), and microtubule-associated protein tau (*MAPT*) in AD [11]. Especially, BIN1 plays a pivotal role in modulating proinflammatory activation and gene expression associated with neurodegeneration in primary mouse microglia [12]. Proliferated and activated microglia concentrated around amyloid plaques are another prominent histopathological change in AD [13].

**Figure 1:**
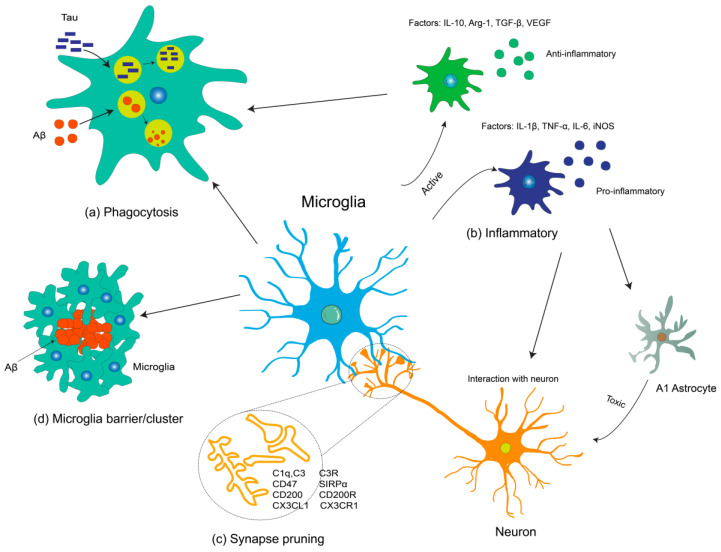
Microglial functions in AD. (**a**) Microglia play a protective role in the brain by removing harmful Aβ and tau through phagocytosis. (**b**) In response to changes in the surrounding microenvironment, microglia alter their morphology. Activated microglia can have pro-inflammatory or anti-inflammatory roles by secreting cytokines. Pro-inflammatory cytokines can activate A1 astrocytes, which are toxic to neurons. Microglia interact with neurons directly or indirectly through physical contact, ligand-receptor binding, signaling, and the release of soluble factors, such as TNF-α, IL-1β, BDNF, and IL-10. (**c**) Microglia are involved in synaptic loss. Signaling pathways related to microglia-mediated synaptic pruning are depicted in the figure. (**d**) Microglia migrate and cluster around amyloid plaques, forming a physical barrier to prevent the spread of Aβ. Abbreviations: IL-1β, interleukin-1β; IL-6, interleukin-6; IL-10, interleukin-10; TNF-α, tumor necrosis factor-α; TGF-β, transforming growth factor-β; iNOS, inducible nitric oxide synthase; Arg-1, Arginase 1; VEGF, vascular endothelial growth factor; CD47, cluster of differentiation 47; CX3CR1, CX3C chemokine receptor 1; CX3CL1, CX3C chemokine ligand 1; CD200, cluster of differentiation 200; SIRPα, signal regulatory protein alpha.

In AD, microglia have a “double-edged sword” effect, displaying both neuroprotective and neurotoxic functions depending on the disease stage and contextual factors [14]. During the early stages of AD, activated microglia primarily exert protective effects by eliminating Aβ plaques through phagocytosis and the release of proteases, as well as engulfing dead cell debris [15]. Additionally, they may adopt alternative reactive states to counteract Aβ-induced cytotoxicity toward neighboring neurons or synapses [16] and create a physical barrier that inhibits plaque spread [17]. However, in the later stages of AD, compromised Aβ clearance and tau accumulation impair microglial defense functions. The dysfunction of microglia is continuously stimulated by the abundance and size of plaques, resulting in an overexpression of proinflammatory cytokines that are toxic to nearby astrocytes and neurons (see Figure 1). These findings underscore microglia as potential therapeutic targets for AD. In this review, we initially delve into the pivotal role played by microglia in AD pathogenesis. Subsequently, we focus on therapeutic strategies aimed at enhancing microglial phagocytic function and regulating neuroinflammation, including both molecular-based drugs and natural products.

## 2. Microglia in AD Pathogenesis

Microglia, the resident macrophages of the central neuronal system (CNS), constitute approximately 10–15% of glial cells under normal conditions. These cells serve as innate immune cells and play a crucial role in maintaining the brain homeostasis [18]. Numerous studies have demonstrated that microglia are essential for CNS development and physiological processes throughout the adulthood and aging. In a healthy mature brain, microglia are the most dynamic cells, constantly surveying the parenchyma through their motile processes and responding to changes in the local environment, even in the absence of pathological challenges [19,20]. Functionally, microglia represent heterogeneous cell populations with gene expression profiles that vary by age and region. Their morphology, ultrastructure, and molecular spectrum exhibit dynamism and plasticity, resulting in the coexistence of distinct cell states closely associated with diverse functions [21].

Recent studies utilizing single-cell RNA sequencing (scRNA-seq) and single-nucleus RNA sequencing (snRNA-seq) have unveiled a spectrum of diverse microglial cell states in both healthy and diseased brains [22]. As the brain ages and in age-related neurodegenerative conditions, microglial phenotypes dynamically evolve across different regions and over time. These reactive states include activated response microglia (ARM), disease-associated microglia (DAM), microglial neurodegenerative phenotype (MGnD), lipid droplet-accumulating microglia (LDAM), white matter-associate microglia (WAM), and dark microglia (DM) [23,24].

DAM, which depend on the triggering receptor expressed on myeloid cells 2 (TREM2), are observed in aging, AD, frontotemporal dementia, and amyotrophic lateral sclerosis (ALS). DAM predominantly express markers such as ApoE, transmembrane protein 119 (TMEM119), P2Y purinoceptor 12 (P2RY12), CX3C chemokine receptor 1 (CX3CR1), cystatin 7 (CST7), and Axl [25,26]. These microglia alter their transcriptional features, enhancing interferon feedback genes, lysosomal genes, lipid metabolism-related elements, and external receptors associated with synapse and neuronal injury [25].

The MGnD is characterized by specific markers, including the C-type lectin domain family 7 (Clec7a), Galectin-3 (Lgals3), Glycoprotein-NMB (Gpnmb), integrin alpha X (Itgax), app1, fatty acid-binding protein 5 (Fabp5), and Ccl2. MGnD can be upregulated by apoptotic neurons and relies on the TREM2-ApoE pathway [27]. Interestingly, this microglial subtype exhibits reduced responsiveness to TGFβ signaling [28] and exerts a protective role in the initial response to neuronal damage [29], particularly in aged brains and neurodegenerative diseases, such as AD, multiple sclerosis (MS), and ALS [27].

In an aging brain, LDAM exhibit severe phagocytosis deficits, representing a dysfunctional microglial state. LDAM produce high levels of reactive oxygen species (ROS) and reactive nitrogen species, along with elevated cytokine release (e.g., IL-6, CCL3, and CXCL10). The LDAM phenotype is regulated by genes including RAS-related protein 1b (RAP1B), receptor for activated C kinase1 (RAK1), solute carrier family 33 member 1 (SLC33A1), sorting nexin 17 (SNX17), Niemann-Pick Disease Type C2 (NPC2), and neuronal ceroid lipofuscinosis 3 (NCL3) [30].

WAM are predominantly observed in aged brains and AD. They express markers such as ApoE, CD63, Clec7a, TMEM119, CX3CR1, P2RY12, and colony-stimulating factor 1 receptor (CSF1R) [31]. WAM formation relies on the activation of the TREM2 signaling pathway. Unlike mouse models of AD, where microglia exhibit early ApoE-dependent features resembling WAM genes, aged-brain microglia generate WAM independently from ApoE [32]. WAM produce proinflammatory cytokines and weaken microglial phagocytosis, potentially contributing to synapse loss [33].

**Figure 2:**
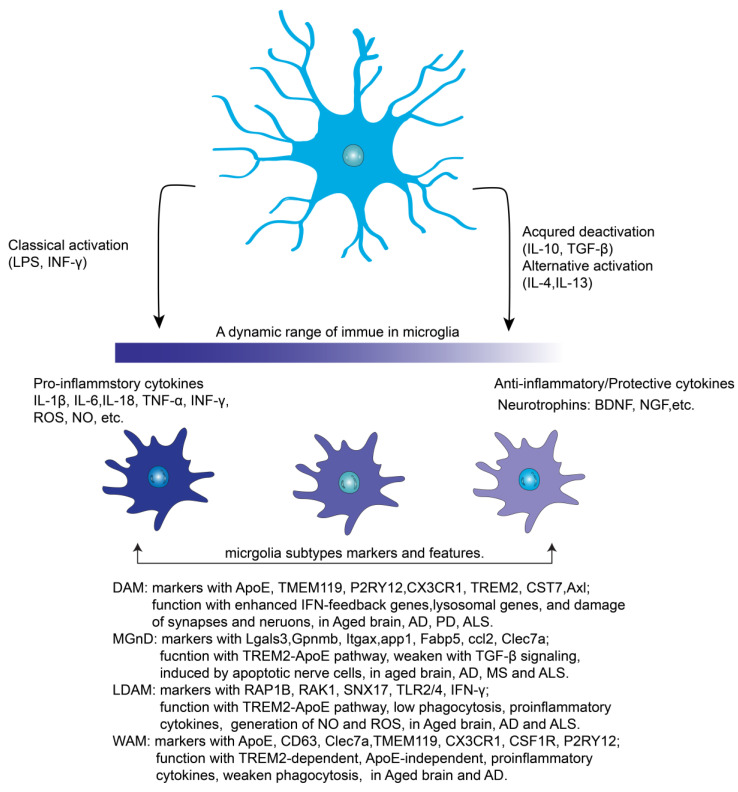
Dynamic range of microglial subtypes in different conditions. Microglia exhibit various reactive states, representing a significant departure from the previously used classical (neurotoxic) and alternative (neuroprotective) activation classifications. This image illustrates the multifunctional roles of microglia within the dynamic immune landscape. Abbreviations: IL-1β, interleukin-1β; IL-6, interleukin-6; IL-10, interleukin-10; IL-18, interleukin-18; IL-4, interleukin-4; TNF-α, tumor necrosis factor-α; TGF-β, transforming growth factor-β; NO, nitric oxide; IL-4, interleukin-4; ROS: reactive oxygen species; INF-γ, Interferon-γ; NGF, nerve growth factor; BDNF, brain-derived neurotrophic factor; DAM, disease associate microglia; MGnD, Microglial neurodegenerative-phenotype; LDAM, lipid-accumulating microglia; WAM, white matter-associate microglia; TMEM119, transmembrane protein 119; P2RY12, P2Y purinoceptor12; CX3CR1, CX3C chemokine receptor 1; TREM2, triggering receptor expressed on myeloid cells 2; CST7, cystatin 7; RAP1B, RAS related protein 1b; RAK1, receptor for activated C kinase1; CD63, cluster of differentiation 63; Clec7a, C-type lectin domain containing 7A; SNX17, sorting nexin 17; Lgals3, Galectin-3; Gpnmb, Glycoprotein-NMB; Fabp5, fatty acid-binding protein 5; ccl2, chemokine (C-C motif) ligand 2; TLR2/4, Toll-like receptor 2/4; CD47, cluster of differentiation 47; CSF1R, colony-stimulating factor 1 receptor; AD, Alzheimer’s disease; PD, Parkinson’s disease; ALS, amyotrophic lateral sclerosis.

Despite the significant transcriptional differences between DAM, MGnD, LDAM, and WAM, they share impairments in phagocytosis and lipid metabolism. This suggests a correlation between the metabolic and functional profiles of these microglial phenotypes [34]. For further details, refer to Figure 2, which illustrates the dynamic range of microglial subtypes in various conditions.

Although GWASs have revealed that most AD risk genes are predominantly or exclusively expressed in microglia, which underscores their significance in AD development [10], the precise role of microglia in AD pathogenesis remains intricate and contradictory. For instance, CSF1R, a single-pass type I membrane protein widely observed in microglia, macrophages, and osteoclasts, plays a crucial role in the development, differentiation, and survival of myeloid lineage cells in the CNS [35]. Selective bioactive blockers of CSF1R, such as PLX3397 (Pexidartinib) and PLX5622, readily cross the blood–brain barrier (BBB) and demonstrate multiple effects on microglial cells. In a study using the tyrosine kinase inhibitor GW2580 to block CSF1R in 6-month-old APP/PS1 mice, cognitive function improved without significantly altering the number of amyloid-β plaques [36]. Further investigations confirmed that CSF1R inhibition significantly reduced amyloid plaques and attenuated AD-like pathology in 5xFAD mice when PLX3397 was administered from 2 months of age and continued for 3 months, or when PLX5622 was administered from 1.5 months of age and continued for 5 months [37,38]. Interestingly, when PLX3397 was administered at a later age (10 months), one month of chronic microglial deletion inhibited neuronal loss and suppressed microglia-related inflammation, with no effect on Aβ pathology burden in 5xFAD mice [39]. Another key player is CX3CR1, a G-protein-coupled receptor belonging to the CXC chemokine family. CX3CR1 interacts with its ligand CX3CL1 (known as Fractalkine or neurokinin), which regulates microglial inflammatory responses. Deficiency in CX3CR1 reduces neuroinflammation and ameliorates amyloid pathology in transgenic AD mice, concurrently reducing inflammatory factors [40,41]. However, conflicting studies suggest that CX3CR1 deletion does not impact amyloid pathology but exacerbates tau pathology and impairs memory retention [42,43]. These findings may be attributed to the different microglial states and the observed stages in animal models. Microglia exhibit a dual nature: they can restrict Aβ and tau propagation by phagocytosing these proteins, while also contributing to the pathological progression of AD by accelerating their dissemination [18,22].

Tauopathy represents another pivotal neuropathological feature of AD pathology. Growing research has demonstrated a close association between activated microglia and tau pathology. Oligomeric and fibrillar forms of tau stimulate morphological alterations in microglia and increase IL-6 expression in these cells [44]. Microglia-related exosomes participated in the propagation of tau in a modified P301L tau mouse model. In this model, AAV-viral transduced expression leads to rapid tau spreading from the entorhinal cortex to the dentate gyrus (DG) of the hippocampus [45]. Depleting or inhibiting microglia, particularly by reducing exosome secretion using small molecules, significantly suppresses tau propagation [46]. These findings are further supported by other studies showing that extracellular vesicles containing phosphorylated tau, secreted by activated microglia, facilitate tau spreading and contribute to the progression of tauopathy in AD transgenic mice.

Recent evidence highlights that activated microglia not only directly release proinflammatory factors [47], but also induce A1 astrocytes by secreting TNF-α, IL-1β, and C1q, contributing to neuronal death in AD pathogenesis [48]. These proinflammatory molecules, including TNF-α and IL-1β, play multifunctional roles in modulating synaptic plasticity [49,50]. For instance, increased IL-1β expression is observed during long-term potentiation (LTP). Blocking the IL-1β receptor in mice leads to delayed escape platform reaching in the Morris water maze and impairs LTP formation in the hippocampus, suggesting IL-1β’s involvement in memory processes [51,52]. Additionally, TNF-α triggers the JNK and NF-κB p65 signaling pathways, promoting iNOS expression and ultimately resulting in neuronal apoptosis and death [50,53].

Overactivated microglia, triggered by complement signaling, excessively engulf functional synapses, leading to impaired synaptic circuits and deficits in LTP [18,54]. Soluble Aβ oligomers, in conjunction with C1q, exhibit toxic effects on synapses and hippocampal LTP. In adult brains, microglia reduce synaptic contents in a CR3-dependent process when exposed to soluble Aβ oligomers [54,55]. Complement C3 deficiency enhances synaptic plasticity in the hippocampus of APP/PS1 mice [56]. Chronic neuroinflammation associated with Aβ plaques drives progressive synaptic dysfunction and memory decline. Activated microglia may also eliminate functional synapses while clearing toxic proteins, including accumulated Aβ [57]. Microglia play a crucial role in elucidating the association between amyloid load and synaptic loss in AD [58]. Dysfunctional microglia directly contribute to neurological alterations in AD by eliminating synapses and exacerbating cognitive decline. Pharmacologically attenuating microglial activation, without affecting Aβ production, can restore synaptic plasticity and cognitive function [39]. Overall, understanding the intricate relationship between amyloid load, synaptic loss, and microglial function is crucial for developing therapeutic strategies in AD.

## 3. Microglia-Related Treatment for AD

With the FDA’s approval of Aducanumab in 2021 [59] and Lecanemab in 2023 [60], mounting evidence demonstrating that reductions in amyloid plaques observed through PET scans are associated with a deceleration in cognitive decline. Anti-Aβ immunotherapy represents another facet of the interplay between microglia and Aβ plaques. During anti-Aβ immunotherapy, microglia are activated through FcγR-mediated antibody-Aβ complex phagocytosis, participating in the Aβ clearance process [61]. This mechanism reduces Aβ plaque formation and neurotoxicity. Ongoing clinical trials investigating microglia-related drugs will provide further evidence for AD immunotherapy.

Drawing from Cummings’ article [5] and the data from clinicaltrials.gov (as of 1 January 2024, encompassing all clinical trials evaluating drug therapies for AD), our focus has been directed toward microglia-related research. Currently, three drugs are undergoing phase II clinical trials: AL002 (a TREM2-activating antibody), Canakinumab (a monoclonal antibody targeting IL-1β), and Pegipanermin (XPro1595), a novel selective TNF-α inhibitor. In the following sessions, we will discuss the modulation of microglial state for the treatment of AD, focusing on two aspects: (1) enhancing microglial phagocytic function and (2) reducing microglia-mediated inflammatory responses.

### 3.1. Enhancing Microglial Function

Microglia, often referred to as the scavenger cells of the brain, play a crucial role in engulfing cellular debris and removing toxic and abnormal proteins, including aggregated Aβ, through phagocytosis. However, in AD brains, microglia exhibit altered branch lengths and large soma, demonstrating alternative phenotypes, such as DAM, MGnD, LDAM, and WAM (as shown in Figure 2). These cells exhibit compromised phagocytic function, impacting their ability to reduce amyloid load in the brain. GWASs have identified innate immune genes preferentially expressed in microglia, including *TREM2*, complement component (3b/4b) receptor 1 (*CR1*), inositol polyphosphate-5-phosphatase (*INPP5D*), spleen focus forming virus (*SFFV*), proviral integration oncogene 1 (*SPI1*), ATP-binding cassette, sub-family A (ABC1), member 7 (*ABCA7*), SH3GL interacting endocytic adaptor 1 (*SGIP1*), BIN1, phosphatidylinositol binding clathrin assembly protein (*PICALM*), CD2-associated protein (*CD2AP*), and the *MS4A* gene cluster [62,63]. These susceptible genes play a critical role in increasing disease risk and provide potential specific pathways and targets for disease pathobiology. Regulating microglial morphology and enhancing phagocytic capacity represent promising targets for AD treatment. Treatments aimed at enhancing microglial phagocytic function are summarized in Table 1.

The most extensively investigated gene is TREM2. TREM2 is a cell surface receptor expressed on microglia, and plays a critical role in regulating inflammation, enhancing phagocytosis, and promoting microglial survival. Upon activation, TREM2 triggers an ApoE-dependent signaling pathway that shifts microglia from a homeostatic state to a neurodegenerative phenotype, resulting in increased inflammatory cytokine levels and accelerated AD pathology [27]. TREM2 exhibits a high affinity for soluble Aβ oligomers and associates with the adaptor protein DNAX-activating protein 12 (DAP12), forming the TREM2-DAP12 complex. The intracellular domain of DAP12 contains an immunoreceptor tyrosine-based activation motif (ITAM), which becomes phosphorylated upon TREM2 ligand binding. This phosphorylation activates spleen tyrosine kinase (Syk), initiating a cascade of signaling events, including phospholipase Cγ (PLCγ) activation, calcium release, and the activation of pathways such as MAPK and PI3K [64]. In mouse models, TREM2 deletion or expression of the TREM2 R47H variant prevents microglial clustering around Aβ plaques, attenuates microgliosis, and facilitates Aβ and tau seeding and spreading near neuritic plaques, underscoring the key role of TREM2 and microglia in limiting peri-plaque tau pathologies [65,66,67]. Overexpression of TREM2 reprograms microglial responsiveness, enhancing process ramification and phagocytic marker expression in plaque-associated microglia, ultimately ameliorating amyloid pathology and cognitive deficits [68]. Soluble TREM2 enhances microglial proliferation and migration toward amyloid plaques, promoting the phagocytic degradation of Aβ [69]. Elevated TREM2 levels in mouse microglia significantly increase phagocytosis-related proteins and improve neuropathology, suggesting that boosting the removal of cellular debris and potentially clearing extracellular Aβ plaques may be achieved through a TREM2-agonistic antibody [68]. Agonistic anti-TREM2 antibodies have been developed to enhance these protective functions in patients with intact TREM2 alleles [70]. AL002c, a specific TREM2 antibody, reduces filamentous plaques and neurite dystrophy, improves cognitive behavior, and attenuates microglial inflammatory responses [71]. It is currently being investigated in two phase II clinical studies (NCT04592874 and NCT05744401) [5]. Another TREM2 agonist, 4D9, enhances microglial clearance of myelin debris and Aβ in vitro, reduces amyloid pathology, and promotes microglial phagocytosis in transgenic AD mice [72]. Microglia-derived soluble TREM2 binds to transgelin-2 (TG2) expressed on neurons, inducing RhoA phosphorylation at position S188 and inactivating the RhoA-rock-GSK3β pathway, thereby ameliorating tau phosphorylation [73]. Overexpression of soluble TREM2 or administration of the active peptide rescues tau pathology and behavioral defects in tau P301S transgenic mice. The sTREM2-TG2 interaction mediates the cross-talk between microglia and neurons. Furthermore, the TREM2 function can be restored by replacing mutant microglia throughout the brain with circulation-derived myeloid cells (CDMCs) following hematopoietic cell transplantation in 5xFAD mice, resulting in ameliorated amyloid pathology. CDMCs restore DAM gene expression with Syk signaling-dependent transcription [74]. However, conflicting reports suggest that TREM2 deficiency significantly decreases tau-stimulated neurodegeneration, attenuates neuroinflammation, and reduces pro-inflammatory cytokine levels in PS19 tauopathy mice [75]. These varying results may be attributed to different animal models and the observed disease stages in understanding the role of TREM2 in AD pathology.

CD33 is another AD-susceptibility gene that encodes a transmembrane glycoprotein widely observed on microglia. In the brains of AD patients, CD33 expression is significantly increased in microglia and closely associated with Aβ aggregation. CD33 expression weakens microglial phagocytosis of Aβ and promotes neuroinflammatory reactions, ultimately facilitating amyloid deposition in transgenic AD mice [76,77]. The deletion of CD33 increases the expression of anti-inflammatory genes, reduces amyloid plaques, and improves cognitive function in AD mice [78]. AL003, a CD33-blocking antibody, has entered the first phase of clinical trials (Clinicaltrials.gov, ID# NCT03822208) [79].

Single nucleotide polymorphisms (SNPs) in the CR1 gene are associated with the risk of AD [80]. Increased copy number variation in CR1 leads to a greater number of C3b-/C4b-binding sites, further linking CR1 to AD risk [81]. CR1 encodes a receptor that binds to complement factors C1q, C3b, and C4b in microglia. Under normal conditions in the CNS, microglial phagocytosis through complement-related signaling pathways removes unnecessary and immature synapses [18]. Notably, synaptic loss occurs in the brains of AD patients and AD model mice. Knockout of complement factors, such as C1q and C3, blocks C3b/CR complement activation, promotes Aβ phagocytosis, inhibits synapse loss, and ameliorates cognitive decline in AD model mice [82].

Granulin (GRN), a secreted multifunctional growth factor, is associated with the onset of late-stage AD. GRN regulates lysosomal biogenesis, inflammation, repair, stress response, aging, and maintenance of neurons and microglia in the mammalian brain [83]. Selective downregulation of PGRN expression in microglia of AD mice leads to increased plaque accumulation and exacerbation of cognitive deficits. Lentivirus-mediated PGRN overexpression reduces amyloid plaque pathology, prevents spatial memory impairments, and preserves hippocampal neurons in AD mice [84].

MicroRNA-155 (miR-155), highly expressed in immune cells, plays a pivotal regulatory role in MGnD [85]. Microglial deletion of miR-155 induces a pre-MGnD activation state through IFN-γ signaling. This phenotypic shift promotes amyloid plaque compaction, reduces neuronal process malnutrition, mitigates plaque-associated synaptic degeneration, and enhances cognitive function. The inhibition of IFN-γ signaling attenuates MGnD-induced phenotypic reversion and impairs microglial phagocytosis [86]. The regulatory mechanism of MGnD mediated by miR-155 and IFN-γ signaling has a beneficial effect on limiting the AD pathology and maintaining cognitive function, suggesting that miR-155 and IFN-γ are potential targets for AD treatment.

Piezo1, a mechanosensitive ion channel [87], is selectively upregulated in microglia associated with Aβ plaques. It can detect the stimulus intensity of Aβ fibrils and induce Ca^2+^ influx, leading to microglial aggregation, phagocytosis, and compaction of Aβ plaques. The absence of Piezo1 from microglia exacerbates Aβ pathology and cognitive decline, while the pharmacological activation of Piezo1 in microglia can reduce brain Aβ load and improve cognitive dysfunction in 5×FAD mice [88]. Therefore, Piezo1 serves as a mechanical sensor for detecting the stiffness of Aβ fibers in microglia and represents a promising therapeutic target for AD [89].

RIPK1, highly expressed in microglia within the human AD brain, inhibits the phagocytic activity of microglia. Pharmacological or genetic inhibition of RIPK1 leads to reduced neuroinflammation, decreased Aβ accumulation in the brain, and improvement in behavioral deficits by enhancing microglial clearance of Aβ [90].

SYK, a non-receptor tyrosine kinase expressed in microglia cells of AD, plays a pivotal regulatory role in the phagocytosis and the acquisition of DAM during demyelinating diseases [91]. The absence of SYK from microglia exacerbates Aβ deposition, aggravated neuropathology, and leads to cognitive deficits in the 5xFAD mice. Moreover, the disruption of the SYK signaling pathway restricts DAM development, modulates the AKT/GSK3β signaling pathway, and impairs Aβ phagocytosis by microglia [92]. The systemic administration of an antibody against CLEC7A, a receptor that directly activates SYK, rescues microglia activation in mice expressing the TREM2R47H allele and attenuates Aβ burden while preserving microglial activation [64,92].

Microglia TAM receptor tyrosine kinases, Axl and Mer, are associated with the pathogenesis of AD [93]. Induction expressions of Axl and Mer in amyloid plaque-associated microglia were coupled with the plaque decoration induced by the TAM ligand Gas6 and its co-ligand phosphatidylserine. TAM-deficient APP/PS1 mice exhibit reduced compact nuclear plaques compared to APP/PS1 mice with normal microglia. The TAM system plays a crucial role in microglia recognition and the phagocytosis of amyloid plaques. TAM-driven microglia phagocytosis facilitated the formation of dense-core plaque [94].

**Table 1:** Microglia-targeted therapies for enhancing microglial phagocytic function.

Targets	Cellular Function in AD	Genetic Manipulations/Pharmacological Interventions	Target/Mechanism	Mechanism of Action	References
CD33	Negative microglia phagocytosis	CD33 knockout; AAV-mediated miRCD33 Inhibitor: P22; Lintuzumab; AL003	Blocked CD33 expression	Promoted Aβ phagocytosis and clearance; decreased neuroinflammatory	[76,78,95,96]
Complement C3	Microglia-mediated synaptic refinement	C3 knockout	Ameliorated C3b/CR3 complement activation	Decreased inflammatory cytokines; promoted Aβ phagocytosis and inhibited synapse loss	[54,56]
GRN, a secreted pleiotropic growth factor	Microglia-mediated phagocytosis	Selectively reducing microglial expression of PGRN; Lentivirus-mediated PGRN overexpression	Exacerbated microglial activation	Impaired phagocytosis; increased plaque load and exacerbated cognitive deficits Lowered plaque load; prevented spatial memory deficits	[84]
MicroRNA-155 (miR155) and IFN-γ	Mediated a protective microglial state	Deletion of miR-155; blocked IFN-γ signaling	Induced a pre-MGnD activation state via IFN-γ signaling Attenuated MGnD induction and microglial phagocytosis	Restricted neurodegenerative pathology and preserved cognitive function	[86]
Piezo1	Microglial mechanosensor of Aβ fibril	Piezo1 deletion; pharmacological activation of Piezo1	Modulated the microglial mechanosensing pathways	Exacerbated Aβ pathology and cognitive decline; ameliorated brain Aβ burden and cognitive impairment	[88]
RIPK1	Microglia-mediated phagocytosis	RIPK1 deletion or Inhibitor	Enhanced the phagocytosis; reduced the inflammatory response	Reduced amyloid burden; the levels of inflammatory cytokines; and memory deficits	[90]
SYK	Regulator of microglia activation and phagocytosis	SYK deletion An antibody against CLEC7A	Restricted microglia phagocytosis; altered AKT/GSK3β-signaling; Directly activates SYK	Exacerbated Aβ deposition; cognitive defects Rescued microglia activation	[64,92]
TAM receptor	TAM-driven microglial phagocytosis	TAM deficient	Reduced microglia detect and engulf Aβ plaques	Developed fewer dense-core plaques	[94]
TREM2	Positive microglia phagocytosis and enclosed to Aβ Activating TG-2, receptor for sTREM2 on neuron	TREM2 overexpression; soluble TREM2; soluble TREM2; agonist: AL002; 4D9. Transplantation of Trem2+/+ CDMC	Reprogramed microglia responsivity; enhanced microglia phagocytosis; sTREM2-TG2 interaction mediates the cross-talk between microglia and neurons; enhanced microglia phagocytosis; restores microglial function with Syk signaling-dependent transcription	Ameliorates amyloid pathology and behavioral deficits; enhanced Aβ clearance and rescued spatial memory; ameliorated tau phosphorylation and cognitive deficits; promote Aβ uptake and clearance, decrease neuroinflammatory; ameliorates amyloid pathology	[68,69,71,72,73,74]

Abbreviations used: CX3CR1: CX3C motif chemokine receptor 1; TAM receptor: Tyro3, Axl, and Mer tyrosine kinases receptor; TREM2: triggering receptor expressed on myeloid cells 2; RIPK1: receptor-interacting protein kinase 1; SYK: spleen tyrosine kinase; CDMCs: cardiac-derived mesenchymal-like cells; TG2: transgelin-2; PGRN: progranulin.

### 3.2. Regulation of Neuroinflammation

Numerous studies showed that the activated microglia induced by aggregated Aβ produced pro-inflammatory cytokines and chemokines, such as TNF-α, IL-6, and IL-1β, leading to neurological dysfunction. Targeting the regulation of microglial activation by preventing the pro-inflammation or activation of anti-inflammation pathways may accelerate the exploration of efficient drugs for AD (Table 2).

Non-steroidal anti-inflammatory drugs (NSAIDs) have been shown to reduce neuroinflammation induced by glial cells and ameliorate the amyloid-like pathology in animal models [97]. In transgenic mouse models of AD, treatment with ibuprofen reduced Aβ plaque load and attenuated microglial activation [98]. Other NSAIDs have also been found to reduce Aβ expression by suppressing γ-secretase expression in transgenic AD mice [99]. Similarly, PPAR-γ agonists, such as GFT1803 or pioglitazone, reduced amyloid pathology and decreased gliosis activation [100,101]. However, clinical trials administering NSAIDs, selective COX-2 inhibitors, low-dose prednisone, and aspirin failed to achieve positive outcomes in delaying cognitive decline in mild-to-moderate AD patients [3].

Minocycline, a semisynthetic tetracycline, exhibits various biological actions, including anti-inflammatory, anti-apoptotic, and neuroprotective effects in mouse models of AD [102]. Minocycline treatment reduces p-tau levels and insoluble tau aggregation in mice, prevents Aβ-induced neurotoxicity in primary cortical neurons, and suppresses neuroinflammatory actions while ameliorating memory deficits in tau transgenic AD mice [103,104].

TNF-α, a pleiotropic and pro-inflammatory cytokine, has been implicated in various neurodegenerative disorders, including AD [105]. Elevated TNF-α levels are strongly correlated with the incidence of AD. Numerous studies have demonstrated that individuals with rheumatoid arthritis (RA) and other systemic inflammatory diseases who utilize TNF-α blockers exhibit a reduced risk of developing dementia compared to the general population [106]. Three extensive epidemiological investigations have reported a significant 60 to 70% lower odds ratio (OR) of developing AD among patients treated with Etanercept [107]. Additionally, two small-scale randomized controlled trials (RCTs) have shown cognitive function improvements among AD patients receiving etanercept treatment [107]. The TNF-α inhibitor XENP345 suppresses AD-like pathology and attenuates chronic systemic inflammation in 3xTg-AD mice by blocking the TNF signaling pathway [108]. Treatment with a TNF-α inhibitor, such as thalidomide or 3,6′-dithiothalidomide, reduces neuroinflammation, decreases amyloid precursor protein (APP) processing, Aβ plaque formation, and promotes cognitive function in transgenic mouse models of AD [109,110]. Currently, Pegipanermin (XPro1595), a novel selective TNF inhibitor, is being investigated in phase II clinical trials (NCT05318976; NCT05522387) [5].

The P2X7 receptor (P2X7R), a purinergic receptor, plays a role in inflammation and immunity. Previous studies have shown that P2X7R mediates NLRP3 inflammasome activation, leading to the release of cytokines and chemokines [111]. Elevated P2X7R levels are found in microglia around senile plaques in AD patients and transgenic AD mice [112,113]. P2X7R activation enhances microglial migration to Aβ plaques but reduces their phagocytic capacity [112]. Downregulation or pharmacological inhibition of P2X7R ameliorates amyloid pathology and promotes behavioral alterations in the early and severe stages of AD. Additionally, P2X7R is involved in the cross-talk between degenerative neurons and microglial activation, resulting in the activation of astrocyte and induced neuroinflammation responses [114,115].

In AD pathology, the phagocytosis of Aβ by microglia triggers NLRP3 inflammasome activation, followed with caspase-1 activation and the release of IL-1β. Elevated NLRP3 expression is observed in AD and MCI brains, as well as in AD mice [116]. The deletion of NLRP3 and caspase-1 reduces NLRP3 inflammasome activation, shifts microglial cells toward a neuroprotective state, ameliorates Aβ pathology, and improves cognitive deficits in APP/PS mice [117]. NLRP3 inflammasome inhibitors, such as JC-124 and fenamate, yield beneficial behavioral outcomes and ameliorate amyloid-like pathology in transgenic AD mice [118,119]. Heneka et al. demonstrated that NLRP3 inflammasome deficiency promotes microglial M2 polarization, reduces brain caspase-1 and IL-1β activation, and contributes to improved cognitive function and reduced Aβ plaques [117]. An antibody neutralizing extracellular ASC blocks amyloid pathology and other potential deleterious inflammatory responses [120]. Blocking the IL-1β pathway ameliorates neuroinflammatory reactions in the brain by reducing NF-κB activation, decreasing tau pathology, and partially inhibiting the expression of oligomeric and fibrillar Aβ in 3xTg-AD mice [121]. Canakinumab, a human anti-IL-1β monoclonal antibody explored by Novartis, is planned for phase II clinical trials (NCT04795466) [5].

Following NLRP3 activation, the apoptosis-associated speck-like protein containing a C-terminal caspase recruitment domain (ASC) forms fibrils and recruits caspase-1, leading to the assembly of ASC specks. These specks can be leaked into the extracellular space, via a prion-like transmission, and spread to nearby microglia, activating an inflammation response [120]. The extracellular ASC specks have a high affinity to Aβ, contributing to cross-seed Aβ oligomerization and plaque formation in APP/PS mice [122]. An antibody neutralizing extracellular ASC ameliorates amyloid burden, blocks inflammasome activation, and mitigates potential deleterious inflammatory processes [120].

In APP/PS1 transgenic AD mice, specific TLR4 suppression using the inhibitor TAK242 provided neural protection and promoted M2 microglial polarization, while suppressing pro-inflammatory cytokines [123]. The deletion of Toll-like receptors (TLRs) enhanced microglia-related Aβ phagocytosis and shifted microglia from a pro-inflammatory M1 state to a neuroprotective M2 state in transgenic APP mice [124]. The inhibition of TLR2 activation reduced neuroinflammatory reactions, ameliorated amyloid pathology, and rescued spatial memory in APP/PS1 double transgenic mice [125]. TLR2 deficiency further promoted the microglial switch from a pro-inflammatory M1 state to a neuroprotective M2 state, suggesting that blocking TLR2 might be a potential treatment for AD [126].

Although IL-10 and IL-4 are commonly regarded as anti-inflammatory factors, their roles in AD research present contradictions and conflicts. Knockdown of IL-10 reduced neuroinflammation and Aβ deposition but exacerbated cognitive deficits [127]. Conversely, AAV-mediated IL-10 overexpression increased ApoE expression, impairing glial cell phagocytosis and resulting in Aβ deposition along with worsened cognitive behavior [128]. The overexpression of IL-4 mitigated neuroinflammation, reduced gliosis, and attenuated Aβ deposition [129]; however, another report suggested that IL-4 overexpression suppressed microglia-mediated Aβ clearance, leading to increased deposition [130]. On the other hand, the overexpression of the pro-inflammatory factor IL-6 enhanced microglial phagocytic capacity while reducing Aβ accumulation [131]. As this field rapidly evolves, observations may vary at different time points and with different transgenic models, necessitating caution when analyzing results from AD models involving microglia.

**Table 2:** Microglia-targeted therapies for the regulation of neuroinflammation.

Targets	Cellular Function in AD	Genetic Manipulations/Pharmacological Interventions	Target/Mechanism	Mechanism of Action	References
TNF-α	Exacerbate inflammation	TNF-α AAV-mediated overexpression Antibodies: XENP345, Thalidomide	Enhanced the microglia response Decrease neuroinflammatory	Induced robust glial activation attenuated plaque deposition Reduced Aβ plaques, and inhibited inflammatory cytokines and APP processing	[108,109,110,132]
P2X7 receptor	Exacerbate inflammation	Inhibitor: Brilliant blue G	Reduced neuroinflammatory	Attenuated gliosis; diminished leakiness of blood–brain barrier	[133]
NLRP3	Exacerbate inflammation	NLRP3 knockout; Casp 1 knockout; loss of NLRP3; inhibitor: JC124	Reduced NLRP3 inflammasome activation; decreased the inflammasome	Ameliorated amyloid pathology and skewed microglial cells to an M2 phenotype; reduced Tau pathology; ameliorated the amyloid pathology and improved spatial memory	[117,118,134]
Extracellular ASC speck	Bound to Aβ and cross-seed Aβ	Injection of ASC specks; antibody-neutralizing extracellular ASC	Inflammasome-dependent formation of ASC specks	Blocked amyloid pathology	[120]
IL-1β	Exacerbated inflammation	IL-1β transgenic antibodies: Canakinumab	Enhanced the microglia response; decreased neuroinflammatory	Mediated chronic neuroinflammation and ameliorated amyloid pathology; decreased NF-κB activity and reduced tau pathology	[121,135]
miR-25802	Microglia-mediated neuroinflammation	Overexpression of miR-25802; inhibition of miR-25802	miR-25802/KLF4/NF-κB signaling axis	Aggravated AD-related pathology, including cognitive disability, Aβ deposition, and microglial pro-inflammatory state; ameliorated AD-related pathology, improved spatial memory, and microglial anti-inflammatory state	[136]
TLR2	Exacerbated inflammation	TLR2 knockout	Reduced neuroinflammatory	Shifted M1 microglia to M2 inflammatory activation	[126]
TLR4	Exacerbated inflammation	A loss-of-function TLR4 mutation; Inhibitor: TAK242	Reduced microglial activation Decreased neuroinflammatory	Increased Aβ deposits and exacerbated cognitive deficits Promoted M2 microglial polarization and suppressed inflammatory cytokines	[123,137]
IL-10	Mediated inflammation	IL-10 knockout	Decreased neuroinflammatory	Ameliorated amyloid pathology and promoted cognitive deficits	[127]
IL-4	Mediated inflammation	IL-4 AAV-mediated overexpression	Decreased neuroinflammation; acute suppression of glial clearance mechanisms	Reduced microgliosis; attenuated amyloid pathology	[129]

Abbreviations used: P2X7 receptor: P2X ligand-gated ion channel receptor; NLRP3: nucleotide-ding domain-like receptor protein 3 inflammasome; TLR4: toll-like receptor 4; TLR2: toll-like receptor 2.

## 4. Microglia-Targeted Modulation by Natural Products for the Prevention and Treatment of AD

An increasing number of AD candidate drugs are entering clinical trials, indicating a growing interest and investment from government, advocacy groups, charities, and biotech and pharmaceutical companies in defeating AD [5,138]. Natural products with preventive potential have garnered attention as alternative therapeutic agents against AD. The neuroprotective and anti-neuroinflammatory effects of natural compounds have been explored through preclinical and clinical studies using in vitro and in vivo models (for a comprehensive review, see [139,140]). Antioxidant and anti-inflammatory compounds, including terpenoids, phenolic derivatives, alkaloids, glycosides, and steroidal saponins, have demonstrated potential for ameliorating amyloid-like pathology and improving the behavioral deficits observed in AD [139]. Various natural products play remarkable roles in attenuating Aβ-induced neuroinflammation, improving memory deficits, and regulating microglial status. In this summary, we focus on natural products and their derivatives involved in regulating inflammatory pathways, particularly those studied using microglial cell lines in vitro and/or in vivo AD animal models.

Gallic acid (polyphernols), a histone acetyltransferase inhibitor, suppressed Aβ induced neurotoxicity by inhibiting microglial-mediated neuroinflammation in an AD ICR mice model and inhibited BV2/primary microglia activation in vitro [141]. Geniposide (gardenia) attenuates an Aβ-induced inflammatory response by targeting RAGE-dependent signaling in BV2 microglial cells [142]. Gypenoside (gynostemma, pentaphyllum) attenuated Aβ-induced inflammation in N9 microglial cells by suppressing proinflammatory mediators (iNOS, TNF-α, IL-1β, and IL-6), increasing the expression of anti-inflammatory proteins (IL-10 and Arg-1), as well as promoting the secretions of BDNF and glial cell-derived neurotrophic factor (GDNF) through the regulation of the suppressing cell signaling protein 1 (SOCS1) signal pathway [143].

Lycium barbarum (LB), an upper-class traditional Chinese medicine, has been utilized for two thousand years and has demonstrated significant health and therapeutic effects [144,145]. Lycium barbarum polysaccharide (LBP) attenuated Aβ-induced neurotoxicity in primary cortical neurons [146,147], enhanced neurogenesis, ameliorated amyloid pathology, and improved cognitive functions in AD transgenic mice [148,149]. Our previous studies showed that LBE (LB extract) promoted M2 polarization (anti-inflammatory phenotype), decreased oligomeric Aβ-induced inflammatory reactions in IMG microglial cell line [150], and preserved retinal function via synaptic stabilization in 3xTg-AD mice [145]. Other than the AD research, LBP has shown an anti-inflammatory effect and reduced the expression of proinflammatory cytokines, partly through suppression of the nuclear factor NF-κB pathway in BV2 microglial cells and liver injury models [151,152].

NF-κB, a well-studied transcription factor, is widely expressed and plays a crucial role in regulating gene expression related to inflammation and immunity [153]. When neuronal and microglial cells were challenged with Aβ, neurotoxicity was linked to the activation of NF-κB signaling. NF-κB activation has been detected in the brains of AD patients and boosted the inflammation reaction in the AD pathological process [154]. Aβ-induced inflammatory responses in BV2 microglial cells were attenuated by the application of Berberine (isoquinoline alkaloid) [155], Loganin (Cornus officinalis) [156], Genistein (Soybean isoflavone) [157], Ginsenoside Rg (Panax ginseng) [158,159], Resveratrol (Polyphenol, red wine) [160], and Hydroxysafflor Yellow A (Carthamus tinctorius) [161] through inhibiting NF-κB pathways. The last three agents also inhibited neuroinflammation in various AD mouse models. Xanthoceraside inhibited pro-inflammatory cytokine expression in Abeta25-35/IFN-gamma-stimulated N9 microglial cells through the TLR2 receptor, MyD88, NF-κB, and mitogen-activated protein kinase (MAPK) signaling pathways [162].

## 5. Concluding Remarks and Future Perspectives

Extensive research highlights microglia’s significant role in Alzheimer’s disease (AD). Recent single-cell analyses reveal distinct microglial phenotypes, advancing our understanding of their functions in AD [9]. Microglia play complex and sometimes contradictory roles—they can limit Aβ and tau spread by engulfing these proteins, yet may also accelerate their diffusion, promoting AD progression. Despite the extensive research on advanced AD stages, investigations into preclinical and early stages remain inadequate. Early detection and treatment are crucial due to the lack of curative interventions for this devastating disease [163]. Understanding microglia’s dynamic state and dominant role at different AD stages is essential for precise therapeutic strategies.

To address translatability concerns, we must differentiate microglial activity observed in animal models from that in AD patients [164,165]. While transgenic mouse models dominate the research, their differences from human microglia raise questions about applicability. Novel model systems, such as those derived from human induced pluripotent stem cells [7,166] or chimeric mouse models [167], offer promising solutions.

Ongoing clinical trials investigating microglia-related drugs will yield new evidence for AD immunotherapy. Additionally, natural products with anti-inflammatory and neuroprotective effects present potential targets for AD drug therapy. Further research into the causal relationship between microglia-related neuroinflammation and AD pathogenesis remains an exciting avenue for innovative therapeutic approaches.

## Abbreviation

AD	Alzheimer’s disease
Aβ	amyloid-β
ABCA7	ATP-binding cassette, sub-family A (ABC1), member 7
ALS	amyotrophic lateral sclerosis.
APP	amyloid precursor protein
APOC2	apolipoprotein C 2
ARM	activated response microglia
Arg-1	arginase 1
ASC	apoptosis-associated speck-like protein containing a C-terminal caspase recruitment domain
BBB	blood-brain barrier
BDNF	brain-derived neurotrophic factor
BIN1	bridging integrator 1
BGW	Bayesian genome-wide
ccl2	chemokine (C-C motif) ligand 2
CD63	cluster of differentiation 63
CD2AP	CD2-associated protein
CDMCs	circulation-derived myeloid cells
CD47	cluster of differentiation 47
CD200	cluster of differentiation 200
Clec7a	C-type lectin domain family 7A
CNS	central neuronal system
CR1	complement component (3b/4b) receptor 1
CST7	cystatin 7
CSF1R	Colony-stimulating factor 1 receptor
CX3CR1	CX3C chemokine receptor 1
CX3CL1	CX3C chemokine ligand 1
DAM	disease-associated microglia
DM	dark microglia
Fabp5	Fatty acid-binding protein 5
GDNF	glial cell-derived neurotrophic factor
Gpnmb	Glycoprotein-NMB
GRN	Granulin
GWASs	genome-wide association studies
INF-γ	Interferon-γ
iNOS	inducible nitric oxide synthase
INPP5D	inositol polyphosphate-5-phosphatase
ITAM	immunoreceptor tyrosine-based activation motif
Itgax	integrin alpha X
LBP	Lycium barbarum polysaccharide
LDAM	lipid droplet-accumulating microglia
Lgals3	Galectin-3
MAPT	microtubule-associated protein tau
MGnD	microglial neurodegenerative-phenotype
MS	multiple sclerosis
NCL3	neuronal ceroid lipofuscinosis 3
NGF	nerve growth factor
NPC2	Niemann-Pick Disease Type C2
OR	odds ratio
PICALM	phosphatidylinositol binding clathrin assembly protein
PLCγ	phospholipase C-γ
PLX3397	Pexidartinib
PD	Parkinson’s disease
P2RY12	P2Y purinoceptor 12
RA	rheumatoid arthritis
RAK1	receptor for activated C kinase1
RAP1B	RAS related protein 1b
RCTs	randomized controlled trials
SFFV	spleen focus forming virus
scRNA-seq	single-cell RNA sequencing
SGIP1	SH3GL interacting endocytic adaptor 1
SIRPα	signal regulatory protein alpha
SLC33A1	solute carrier family 33 member 1
SNPs	Single nucleotide polymorphisms
SNX17	sorting nexin 17
snRNA-seq	single nucleus RNA sequencing
SPI1	proviral integration oncogene 1
SOCS1	suppressing cell signaling protein 1
Syk	spleen tyrosine kinase
TLR2/4	Toll-like receptor 2/4
TMEM119	transmembrane protein 119
TNF-α	tumor necrosis factor-α
TREM2	triggering receptor expressed on myeloid cells 2
TWAS	transcriptome-wide association study
VEGF	vascular endothelial growth factor
WAM	white matter-associate microglia
